# Impact of implementing a prioritization process on waiting time for non-scheduled surgeries in a tertiary emergency unit

**DOI:** 10.1016/j.clinsp.2025.100712

**Published:** 2025-06-20

**Authors:** Marcelo Cristiano Rocha, Sergio Henrique Bastos Damous, Rafaela Alkmin Costa, Edivaldo Massazo Utiyama

**Affiliations:** a Faculdade de Medicina da Universidade de São Paulo, São Paulo, SP, Brazil; b Hospital das Clínicas da Faculdade de Medicina da Universidade de São Paulo, São Paulo, SP, Brazil

**Keywords:** Emergency surgery, Triage, Non-elective procedures, Hospital management, operating rooms

## Abstract

**Objectives:**

To evaluate the impact of implementing a structured prioritization process on surgical waiting times and compliance with time-to-intervention targets for non-elective surgeries in a tertiary academic emergency hospital.

**Methods:**

This observational retrospective single-center cohort study compared two periods before and after implementing a prioritization system. All non-elective surgeries performed from June to August 2022 (pre-intervention) and from November 2022 to January 2023 (post-intervention) were analyzed. The intervention included urgency classification, a real-time Kanban dashboard, and daily multidisciplinary scheduling meetings. The primary outcome was the median time from surgical indication until surgical procedure. The secondary outcome included adherence to predefined acceptable waiting time windows. Statistical comparisons were performed using Chi-Square, Fisher's exact, or Mann-Whitney U tests. A significance level of *p* < 0.05 was adopted.

**Results:**

1851 surgeries were analyzed (967 pre-implementation and 884 post-implementation). The median waiting time was significantly reduced from 17h20min to 8h52min (*p* < 0.001). Compliance with acceptable waiting time windows increased from 60.5 % to 77.1 % (OR = 2.205; 95 % CI 1.799–2.701).

**Conclusions:**

Introducing a structured prioritization strategy significantly reduced waiting times and improved adherence to surgical timelines in a high-complexity emergency environment. The model proved feasible, effective, and well-accepted by multidisciplinary teams. The long-term impact of the implementation of the model could be further addressed in future studies.

## Highlights

• A structured prioritization model was implemented for non-elective surgeries in a tertiary academic hospital in Brazil.
• The intervention combined urgency classification, a Kanban dashboard, and daily multidisciplinary meetings.
• Median surgical waiting time dropped from 17h20min to 8h52min after implementation (*p* < 0.001).
• Compliance with acceptable waiting time windows improved from 60.5 % to 77.1 %.
• The model enhanced surgical efficiency and was well-accepted by multidisciplinary teams.

## Introduction

Delays in surgical emergency care remain a critical challenge in health systems worldwide, particularly in high-demand emergency settings in public and private healthcare systems. While elective procedures can often be planned around available resources, non-scheduled surgeries require a timely response and careful coordination. When left unstructured, these urgent demands can result in inefficient operating room usage, prolonged waiting times, and increased morbidity and mortality.

Evidence consistently shows that emergency surgical patients face worse outcomes than those treated electively. Delays beyond 24 hours have been independently associated with higher complication rates and adverse prognosis, especially among vulnerable populations such as the elderly.^1^, ^2^, ^3^, ^4^, ^5^ Despite this, many hospitals lack formal mechanisms to assess urgency and allocate surgical resources accordingly.

Several international frameworks have sought to address this gap by defining surgical urgency and ideal intervention windows. Examples include the National Confidential Enquiry into Patient Outcome and Death (NCEPOD) in the United Kingdom, the Timing of Acute Care Surgery (TACS) by the World Society of Emergency Surgery, and the Traffic Light System proposed by Leppäniemi and Jousela.^6^, ^7^, ^8^, ^9^ These systems have proven effective in improving surgical planning, reducing delays, and minimizing inter-team conflicts.

However, implementing these frameworks in large tertiary institutions remains complex. Facilities like the Hospital das Clínicas da Faculdade de Medicina da Universidade de São Paulo (HCFMUSP) manage hundreds of emergency surgical cases across multiple specialties. In such environments, decision-making is often subjective, decentralized and inconsistent, leading to scheduling conflicts, lack of transparency, and avoidable delays.^10^, ^11^, ^12^

In response to these issues, a structured prioritization model was introduced at HCFMUSP in September 2022. This group has previously described the model.^13^ This model combined urgency-based triage criteria, a real-time digital Kanban board for visualizing cases, structured triage categories with defined time thresholds, daily multidisciplinary briefings, and continuous monitoring. Its goal was to create a transparent and data-driven system for allocating operating room access to non-elective procedures.^13^

This study aims to evaluate the impact of implementing this structured triage and scheduling system on time-to-surgery for non-elective procedures at a tertiary academic hospital, with the hypothesis that such an approach would reduce delays, improve procedural compliance with waiting time windows, and enhance clinical governance in emergency surgical care.

## Methods

### Study design and setting

This was a retrospective observational single-center cohort study conducted at the Emergency Unit of the Central Institute of the Hospital das Clínicas, University of São Paulo Medical School (ICHC—HCFMUSP), a tertiary teaching hospital with high complexity patients and huge surgical volume. The primary objective was to assess the impact of a newly implemented prioritization process on waiting times for non-scheduled surgeries.

Two distinct three-month periods were analyzed: a pre-implementation phase (June 1 to August 31, 2022) and a post-implementation phase (November 1, 2022, to January 31, 2023). The two-month transitional phase (September to October 2022), during which teams were trained and the system was adapted, was excluded to avoid confounding.

This study is in agreement with the STROBE statements.

### Eligibility criteria

All non-elective surgeries recorded in the hospital’s electronic surgical notification system were considered. Each surgical intervention was treated as an independent event; therefore, patients who underwent more than one procedure within the same period contributed multiple entries to the dataset.

### Exclusion criteria

Procedures related to liver transplantation, obstetrics, or performed under non-public healthcare funding sources (i.e., not covered by the Brazilian Unified Health System – SUS) were excluded from the analysis.

### Data collection

Clinical, demographic, and surgical data were retrieved from the institutional electronic health records (MV-PEP) and the Surgical Production Dashboard (PIH ‒ Plataforma de Inteligência Hospitalar), with anonymization before analysis.

Collected variables included age, sex, surgical medical specialty, acceptable maximum waiting time for the procedure (hours), surgical diagnosis, and time from surgical indication to intervention (hours).

### Outcome measures

The primary outcome was the time from surgical indication to the procedure execution. As secondary outcomes, the authors evaluated the proportion of procedures performed within acceptable waiting timeframes, categorized by surgical specialty and severity of the disease.

### Statistical analysis

Data were tabulated using Microsoft Excel 2023 and analyzed using SPSS Statistics version 29.0. Kolmogorov-Smirnov test was used to assess normality: continuous data normally distributed were described as mean ± standard deviation and compared using the Student’s *t*-test; non-normally distributed data were expressed as median (interquartile range) and compared using the Mann-Whitney *U* test; categorical data were reported as counts and percentages and compared using Chi-Square or Fisher’s exact test, as appropriate. A significance level of 0.05 was adopted.

### Sample size estimation

Based on historical data, the average pre-intervention wait time for non-scheduled surgery was 4.58 ± 6.65 days. Assuming a 20 % reduction in this interval with 95 % confidence and 80 % power, a minimum of 761 procedures per group was required. A three-month sample was deemed sufficient to accommodate potential data loss (15 %), given an average monthly surgical volume of 280 non-elective procedures.

### Ethical considerations

The study was approved by the Hospital das Clínicas Ethics Committee (CAPPesq n 76446223.2.0000.0068). Patient informed consent was waived due to the retrospective and anonymized nature of the study.

## Results

### Sample characteristics

A total of 1851 non-scheduled surgical procedures were analyzed, including 967 performed during the pre-Kanban period (June–August 2022) and 884 during the post-Kanban period (November 2022–January 2023). There were no statistically significant differences in patient age (median 49 vs. 50 years; *p* = 0.181) or sex distribution (female: 40.4 % vs. 39.7 %; *p* = 0.749), indicating demographic comparability between periods (Table 1).

**Table 1:** Comparison of demographic characteristics, surgical specialties, and acceptable waiting time limits for surgical procedures (according to surgical diagnosis) between the pre- and post-Kanban periods (São Paulo, HCFMUSP, 2021‒2022).

Characteristic	Pre-Kanban (*n* = 967) Median [p25‒p75], n ( %)	Post-Kanban (*n* = 884) Median [p25‒p75], n ( %)	p-value
**Age (years)**	49 [32–64]	50 [35–65]	0.181
**Sex**			
Female	391 (40.4 %)	351 (39.7 %)	0.786
Male	576 (59.6 %)	533 (60.3 %)	0.786
**Surgical specialty**			0.025
Digestive surgery	34 (3.5 %)	19 (2.2 %)	0.105
General surgery	230 (23.8 %)	180 (20.4 %)	0.086
Plastic surgery	19 (2.0 %)	17 (1.9 %)	1.000
Vascular surgery	151 (15.6 %)	177 (20.0 %)	0.016
Neurosurgery	235 (24.3 %)	233 (26.4 %)	0.336
Ophthalmology	104 (10.8 %)	65 (7.4 %)	0.014
Orthopedics	28 (2.9 %)	22 (2.5 %)	0.692
Otorhinolaryngology	51 (5.3 %)	57 (6.5 %)	0.329
Others	34 (3.5 %)	36 (4.1 %)	0.614
Urology	81 (8.4 %)	78 (8.8 %)	0.795
**Limit waiting time for surgical procedure**			0.642
Up to 1-hour	39 (4.0 %)	36 (4.1 %)	1.000
Between 1–6 h	229 (23.7 %)	211 (23.9 %)	0.968
Between 6–24 h	290 (30.0 %)	292 (33.0 %)	0.174
Between 24–48 h	386 (39.9 %)	330 (37.3 %)	0.274
More than 48 h	23 (2.4 %)	18 (2.0 %)	0.733

The distribution of procedures by surgical specialty varied significantly between periods (*p* = 0.025). Vascular surgeries increased from 15.6 % to 20.0 %, while ophthalmologic procedures decreased from 10.7 % to 7.4 %. The majority of surgical cases in both periods were classified as needing intervention within 24 to 48 hours (39.9 % pre-Kanban vs. 37.3 % post-Kanban, *p* = 0.274), followed by those requiring surgery within 6 to 24 hours (30.0 % vs. 33.0 %, *p* = 0.174). Procedures requiring intervention within 24 hours represented 57.7 % of all surgeries in the pre-Kanban period and 61.0 % in the post-Kanban period (*p* = 0.167), as presented in Table 1.

### Outcomes

The median time between surgical indication and the surgical procedure decreased significantly from 17:20 (8:50–31:40) in the pre-implementation period to 8:52 (4:33–16:58) in the post-implementation period (*p* < 0.001).

When categorizing the surgical procedures by surgical specialties, there was a statistically significant reduction in the waiting time for procedures for the Neurosurgery, Ophthalmology, and Plastic Surgery groups (Table 2), while other surgical specialties, including vascular surgery, urology, and general surgery, also showed favorable trends, though not statistically significant. No surgical specialty presented an increase in waiting time for surgical procedures.

**Table 2:** Comparison of waiting time for surgical procedures between the pre- and post-Kanban periods, according to surgical specialty (São Paulo, HCFMUSP, 2021‒2022).

	Pre-Kanban (*n* = 967)	Post-Kanban (*n* = 884)	p
**Surgical Specialty**	**Median [p25‒p75]**	**Median [p25‒p75]**	
Digestive Surgery	21:51 [3:50 – 38:11]	4:56 [2:32 – 19:54]	0.170
General Surgery	11:49 [1:36 – 40:22]	6:59 [1:44 – 22:46]	0.067
Plastic Surgery	24:39 [4:54 – 83:30]	3:58 [2:15 – 16:34]	0.011
Vascular Surgery	22:39 [3:38 – 118:22]	17:30 [4:19 – 58:56]	0.421
Neurosurgery	11:21 [2:29 – 59:33]	4:22 [1:30 – 19:48]	<0.001
Ophthalmology	25:38 [14:09 – 48:53]	12:43 [5:58 – 19:12]	<0.001
Orthopedics	6:20 [2:20 – 18:29]	3:37 [1:55 – 8:00]	0.207
Otorhinolaryngology	9:42 [4:28 – 30:38]	7:57 [3:52 – 18:19]	0.112
Urology	47:44 [5:54 – 115:05]	21:55 [5:09 – 42:52]	0.056
Others	45:33 [8:18 – 74:52]	13:59 [3:57 – 47:19]	0.039
**Overall**	**17:20 [8:50 - 31:40]**	**8:52 [4:33 - 16:58]**	***p*<0.001**

The authors further evaluated the attendance of the actual waiting time for the procedure to the pre-established acceptable waiting time windows, categorized by surgical specialty and the severity of surgical diagnosis.

The overall proportion of surgeries performed within the acceptable timeframe increased from 60.5 % to 77.1 % following the intervention (OR = 2.205; 95 % CI 1.799–2.701). Among surgical specialties, this attendance increased within every surgical group, except for the Orthopedics and the others, in which the difference did not reach statistical significance (Table 3).

**Table 3:** Attendance of the actual waiting time to the pre-established acceptable waiting times for surgical procedures, before and after the implementation of Kanban, according to surgical specialty (São Paulo, HCFMUSP, 2021‒2022).

Surgical Specialty	Pre-Kanban (*n* = 967)	Post-Kanban (*n* = 884)	OR (95 % CI)
Digestive System Surgery	16 (47.1 %)	16 (84.2 %)	6.000 (1.472 – 24.454)
General Surgery	153 (66.5 %)	143 (79.4 %)	1.945 (1.236 – 3.061)
Plastic Surgery	9 (47.4 %)	17 (100 %)	2.889 (1.703 – 4.900)
Vascular Surgery	82 (54.3 %)	120 (67.8 %)	1.772 (1.130 – 2.776)
Neurosurgery	145 (61.7 %)	182 (78.1 %)	2.215 (1.474 – 3.327)
Ophthalmology	68 (65.4 %)	58 (89.2 %)	4.387 (1.816 – 10.598)
Orthopedics	22 (78.6 %)	20 (90.9 %)	2.727 (0.493 – 15.095)
Otorhinolaryngology	35 (68.6 %)	49 (86.0 %)	2.800 (1.080 – 7.263)
Urology	38 (46.9 %)	51 (65.4 %)	2.137 (1.128 – 4.049)
Others	17 (50.0 %)	26 (72.2 %)	2.600 (0.964 – 7.010)
**Overall**	**585 (60.5 %)**	**681 (77.1 %)**	**2.205 (1.799 ‒ 2.701)**

**Fig. 1:**
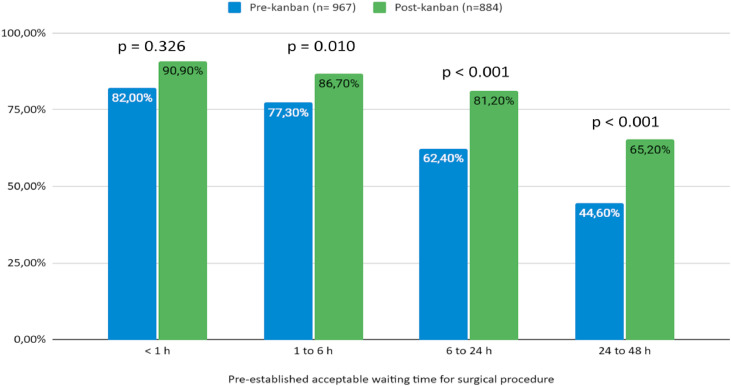
Attendance of the actual waiting time to the pre-established acceptable waiting times for surgical procedures, before and after the implementation of Kanban (São Paulo, HCFMUSP, 2021‒2022).

According to the severity of surgical diagnosis categories, represented by the acceptable waiting time window for the surgical procedure, there was a statistically significant improvement for those surgeries expected to occur within 1h to 6 h, 6h to 24 h, and 24h to 48 h after surgical indication (Fig. 1). For more urgent categories, namely < 1 hour, despite the apparent improvement, there was no significant difference, possibly due to pre-existing rapid response protocols already in place for critical cases.

## Discussion

This study assessed the effects of implementing a structured prioritization model on waiting times and compliance with urgency-based targets for non-scheduled surgeries in a tertiary emergency hospital. The findings demonstrate a substantial improvement in surgical timeliness, reflected by a nearly 50 % reduction in median time to procedure and a marked increase in the proportion of operations performed within recommended time frames. These results align with international evidence supporting the use of structured prioritization and triage systems to improve outcomes in emergency surgical care.

The greatest impact was observed among intermediate urgency categories ‒ particularly procedures expected within 6 to 24 and 24 to 48 hours ‒ where delays are more likely to result from organizational factors rather than clinical instability. By introducing urgency stratification, daily interdisciplinary planning meetings, and a shared real-time Kanban dashboard, the model addressed longstanding bottlenecks in surgical scheduling. These interventions enhanced the visibility of pending cases, improved communication among specialties, and allowed for a more deliberate allocation of operating room access.

Notably, no significant changes were observed in the most urgent category (< 1 hour), which likely reflects the existence of pre-established rapid-response protocols for life-threatening emergencies. This pattern is consistent with prior studies suggesting that the benefits of prioritization models are more evident in cases where delays are avoidable and operational.^10^, ^11^, ^12^, ^13^, ^14^ In other words, structured prioritization appears most effective where decisions can be optimized through coordination rather than urgency alone.

The model also promoted measurable improvements across several surgical specialties. Plastic surgery and digestive surgery showed particularly striking gains in adherence to time targets, with compliance rates increasing from 47.4 % to 100 % and from 47.1 % to 84.2 %, respectively. Similar trends were observed in ophthalmology and other disciplines. These outcomes underscore the broader utility of structured triage not only in expediting care but also in fostering a shared sense of accountability among teams ‒ a critical element in high-volume emergency settings where prioritization often depends on real-time negotiation and resource sharing.

In addition to reductions in median waiting times, the proportion of surgeries performed within the ideal timeframe increased significantly from 60.5 % to 77.1 %. This result further supports the hypothesis that governance tools, such as urgency-based categorization and shared visual dashboards, can enhance the responsiveness and equity of surgical systems. These strategies are especially relevant in tertiary hospitals with multiple overlapping specialties and constrained resources, where subjective prioritization frequently contributes to delays, dissatisfaction, and suboptimal outcomes.

The present findings are consistent with international frameworks such as the NCEPOD classification (UK), the World Society of Emergency Surgery’s TACS model, and traffic-light coding systems, which have all shown benefits in harmonizing surgical response across specialties. Importantly, the model evaluated in this study was developed and implemented within a large, resource-constrained public hospital, suggesting that such strategies are feasible even in complex real-world settings. The design of the intervention emphasized accessibility and transparency, relying on manual but standardized tools ‒ Kanban boards, urgency codes, and daily briefings ‒ to promote participation across disciplines.^6^, ^7^, ^8^

Nevertheless, the study has limitations. Its retrospective and single-center design restricts generalizability, and long-term patient outcomes were not assessed. While the intervention proved operationally successful and was well accepted by clinical teams, it remains dependent on human input and sustained engagement. Future iterations may benefit from integration with digital health records, automated classification, or predictive analytics capable of supporting real-time triage. These enhancements could further increase efficiency while reducing the cognitive and logistical burden on healthcare teams.

Despite these limitations, the intervention demonstrated strong potential as a scalable and low-cost solution to a widespread problem in emergency surgical care. By reducing unwarranted delays and increasing compliance with recommended timeframes, the model directly addressed a critical determinant of outcome in non-elective surgery. Its emphasis on clarity, shared responsibility, and timely access to care offers a reproducible blueprint for similar institutions seeking to improve emergency surgical logistics.

## Conclusion

The implementation of a structured prioritization model for non-scheduled surgical procedures, supported by urgency classification, a real-time shared dashboard, and daily interdisciplinary coordination, led to significant reductions in surgical wait times in a high-complexity tertiary hospital. These findings support the broader adoption of structured prioritization strategies in emergency surgery. The study's limitations ‒ including its retrospective, single-center design and the absence of long-term patient outcome data ‒ must be acknowledged. Further studies are recommended to evaluate the model’s scalability, sustainability, and clinical impact across diverse healthcare environments.

## CRediT authorship contribution statement

**Marcelo Cristiano Rocha:** Conceptualization, Data curation, Formal analysis, Investigation, Methodology, Project administration, Resources, Validation, Visualization, Writing – original draft. **Sergio Henrique Bastos Damous:** Supervision, Validation, Visualization, Writing – review & editing. **Rafaela Alkmin Costa:** Conceptualization, Formal analysis, Methodology, Supervision, Validation, Visualization, Writing – review & editing. **Edivaldo Massazo Utiyama:** Conceptualization, Formal analysis, Methodology, Supervision, Validation, Visualization, Writing – review & editing.

## Declaration of competing interest

The authors declare no conflicts of interest.
